# Responses of metastatic primary fallopian tube carcinoma to pembrolizumab and nab-paclitaxel: A case report

**DOI:** 10.1097/MD.0000000000021203

**Published:** 2020-07-10

**Authors:** Jian Jiang, Zhi-peng Chen, Hui-ping Zhu, Yong-qin Zhang, Xiao-lan Qian, Min Zhang, Chen Ni, Yun Zuo

**Affiliations:** Department of Oncology, The Affiliated Zhangjiagang Hospital of Soochow University, Suzhou, China.

**Keywords:** case report, chemotherapy, immunotherapy, nab-paclitaxel, pembrolizumab, primary fallopian tube carcinoma

## Abstract

**Rationale:**

Primary fallopian tube carcinoma (PFTC) is an extremely rare but invasive malignancy with a dismal prognosis. Very few data exist on the salvage treatment for patients with PFTC. Here we report a case showing an impressive response to immunotherapy combined with chemotherapy, which have never been reported before on patients with metastatic PFTC.

**Patient concerns:**

A 42-year-old woman, who was diagnosed with PFTC in 2010, had been failed of multiple systemic therapies and antiangiogenic therapy because of the disease recurrence and progression.

**Diagnosis:**

Metastatic primary fallopian tube carcinoma.

**Interventions:**

The patient underwent surgery in May 2010 and had multi-line chemotherapies plus an anti-vascular endothelial growth factor (anti-VEGF) monoclonal antibody for about 9 years. Due to treatment failure the patient accepted the immunotherapy with the checkpoint inhibitor, pembrolizumab, combined with nab-paclitaxel from December 2018 to April 2019.

**Outcomes:**

The patient showed a complete response after 6 cycles^,^ treatment. Thus far, the patient is taking pembrolizumab as maintenance and remains in good health.

**Lessons:**

Pembrolizumab combined with chemotherapy for treatment of PFTC may provide a positive antitumor effect in multiple metastatic lesions, but more clinical evidence is needed to confirm the efficacy and safety.

## 1 Introduction

Primary fallopian tube carcinoma (PFTC) is extremely rare and its incidence rate accounts from 0.14% to 1.8% of all gynecological malignancies.^[1]^ In the United States the incidence rate was 0.36 to 0.41 per 100,000 women annually in 2017.^[2]^ A large amount of evidence has confirmed that most epithelial ovarian cancers (EOC) are of fallopian tube origin. Therefore, the incidence of PFTC may be underestimated.^[3,4]^ It occurs in a wide age range from 19 to 80 with a median age of 52 years.^[5]^ PFTC is often misdiagnosed as ovarian carcinoma before laparotomy due to the similarities in clinical and pathological features.^[6]^ PFTC that is more advanced at diagnosis would lead to an unfavorable prognosis.^[7]^

The most common clinical symptoms of PFTC include abdominal pain, serosanguinous vaginal discharge, and pelvic masses, which is called Latzko triad.^[8]^ Histologically serous adenocarcinoma accounts for >90% of all common types.^[9]^ PFTC mainly spreads to the abdominopelvic cavity and adjacent structures such as uterus and ovary and also can disseminate to other metastasis sites by lymphatic or hematogenous routes.^[10]^

Surgery is the primary treatment for PFTC. Adjuvant chemotherapy is considered effective, in view of the mode of lymphatic and hematogenous metastasis for this cancer. A platinum compound combined with paclitaxel is the standard chemotherapy in the treatment of PFTC, identical to ovarian cancer patients.^[11]^ Due to its low incidence and poor prognosis, salvage treatment for patients with PFTC and related efficacy is rarely reported. Programmed death ligand 1 (PD-L1), an immune checkpoint receptor, which is overexpressed in a series of human tumors in order to aid escape from the immune system via cell programmed death-1 (PD-1) signaling. These signaling pathways may represent new treatment choices for PFTC. To date, there is no case report on PFTC for any relevant treatment options.

Here we presented a patient with metastasized fallopian tube carcinoma who had multi-line chemotherapies plus an anti-VEGF monoclonal antibody, with a remarkable clinical response to the immune checkpoint inhibitor, pembrolizumab, and chemotherapy drug nab-paclitaxel. We present the following case in accordance with the CARE Guideline.

## 2 Case report

A 42-year-old woman presented to our institution due to abdominal pain in January 2010. A computed tomography (CT) scan revealed multiple masses in the pelvic cavity, which were considered to be malignant tumors. Serum CA125 levels were >110 IU/mL. The patient had undergone a surgery in May 2010, including total hysterectomy, bilateral adnexectomy, greater omentectomy, and appendectomy. Pathologic analysis revealed that the tumor was comprised of serous papillary adenocarcinoma component with moderate differentiation in the right fallopian tube. Tumor was found infiltrating the entire wall of fallopian tube and invading the ovary. Metastatic nodules (2 cm in diameter) were observed in the uterine serosa and greater omentum.

From May to December 2010, the patient received paclitaxel liposome (135 mg/m^2^) and cisplatin (75 mg/m^2^) every 3 weeks for 8 cycles, and was in stable condition at 3-year follow-up. In October 2013, the patient suffered from abdominal pain with a significant rise in CA125 levels. Diagnostic imaging with positron emission tomography-computed tomography (PET-CT) demonstrated disease progression in surgery area, retroperitoneal lymph nodes, and spleen. Then she was treated with paclitaxel liposome (135 mg/m^2^) and carboplatin (AUC 5 mg/mL·min) for 6 cycles, in combination with the antiangiogenic therapy bevacizumab (7.5 mg/kg), but had disease progression after 1 year.

From May 2015 to January 2016, the patient was treated with nab-paclitaxel (260 mg/m^2^) and nedaplatin (80 mg/m^2^), as well as bevacizumab (7.5 mg/kg). After 7 cycles of therapy, a complete response was reached according to the Response Evaluation Criteria in Solid Tumors 1.0. Unfortunately, after 9 months, PET-CT revealed multiple metastases in the liver, supraclavicular, and retroperitoneal lymph nodes.

**Figure 1:**
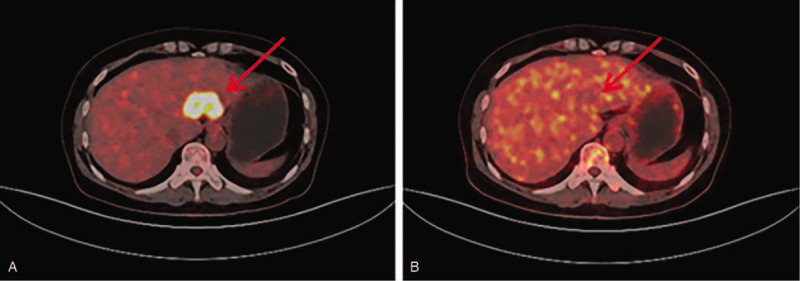
PET-CT showed metastasis in the left liver. (A) Before treatment with pembrolizumab and nab-paclitaxel, the metastasis was 4 cm in diameter. (As indicated by the red arrow, the same below). (B) After 6 cycles of pembrolizumab and nab-paclitaxel, the metastasis was nearly dismissed. PET-CT = positron emission tomography-computed tomography.

**Figure 2:**
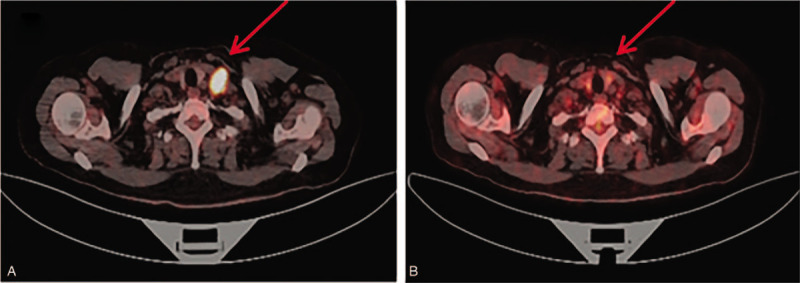
PET-CT showed lymph node metastasis in the left supraclavicular fossa. (A) Before treatment with pembrolizumab and nab-paclitaxel, the metastasis was 1.6 cm in short diameter. (B) After 6 cycles of pembrolizumab and nab-paclitaxel, the metastasis was nearly dismissed. PET-CT = positron emission tomography-computed tomography.

**Figure 3:**
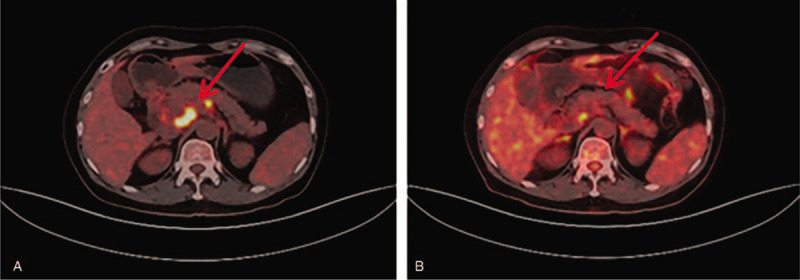
PET-CT showed multiple lymph node metastases in the retroperitoneum. (A) Before treatment with pembrolizumab and nab-paclitaxel, the largest lymph node was 1.6 cm in short diameter. (B) After 6 cycles of pembrolizumab and nab-paclitaxel, the metastases were mostly dismissed. PET-CT = positron emission tomography-computed tomography.

**Figure 4:**
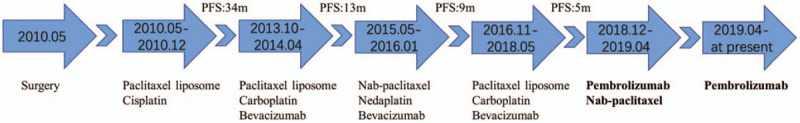
Treatment history. m = month, PFS = progression-free survival.

From November 2016 through May 2018, the patient was intravenously administrated intermittent of paclitaxel liposome (135 mg/m^2^) and carboplatin (area under the curve [AUC] 5 mg/mL·min) for 14 times. Since disease progressed again in November 2018, microsatellite instability status and BRCA 1/2 mutation were determined by next generation sequencing. The results demonstrated microsatellite instability-high (MSI-H) and BRCA1/2 negative. After enough communicating with the patient about adverse and unanticipated events, she wanted to try immunotherapy. The patient was then treated with pembrolizumab (200 mg every 3 weeks) and nab-paclitaxel (260 mg/m^2^) from December 2018. After 6 cycles of therapy, in April 2019, PET/CT revealed complete response in the left liver (Fig. 1), as well as in the left supraclavicular and retroperitoneal lymph nodes (Figs. 2 and 3). Now the patient was transitioned to maintenance pembrolizumab (200 mg every 3 weeks). Please refer to Fig. 4 for the full treatment history.

## 3 Discussion

Over the past few decades, several studies demonstrated an increased incidence of PFTC due to the improvements in diagnostic practices and identification of pathological processes.^[12,13]^ The overall survival (OS) rate for patients with PFTC ranges between 22% and 57%.^[14]^ Stage and residual tumor after initial surgery are important prognostic factors.^[9]^

Since the low incidence of PFTC, large prospectively controlled trials of adjuvant therapy are difficult to conduct. Available therapeutic strategies for PFTC follow guidelines used in EOC. Surgery is the first treatment of choice for patients with PFTC. Due to the high risk of metastases, adjuvant therapy seems to have strong rationale. Currently, platinum/taxane combination chemotherapy is thought to be the standard treatment, especially for patients with advanced PFTC.^[5]^ While the second-line treatment choice is based on the time interval from the finishing of previous platinum-based chemotherapy to the date of disease recurrence. Patients who relapse >6 months are considered platinum-sensitive and should be treated with platinum-based combination chemotherapy. Whereas patients who relapse within 6 months or progress on first-line treatment are considered as having platinum-resistant or platinum-refractory disease. For these patients, non-platinum-based chemotherapy can be selected, including liposomal doxorubicin, gemcitabine, topotecan, paclitaxel, or etoposide. Bevacizumab, as the first targeted therapy in EOC, has been approved by the Food and Drug Administration (FDA) in November 2014 based on 2 phase III trials, which showed a statistically significant improvement in progression-free survival (PFS) and objective response rate (ORR) by adding bevacizumab to chemotherapy but no difference in OS.^[15,16]^ Currently, fewer studies have reported standard second-line or salvage therapeutic regimens in the safety or efficacy for recurrent diseases.

Nab-paclitaxel is a cytotoxic drug which loads paclitaxel into human serum albumin. In recent studies, nab-paclitaxel had shown significant clinical efficacy in recurrent or platinum-resistant EOC with an acceptable side effect.^[17]^ The National Comprehensive Cancer Network (NCCN) has added nab-paclitaxel as option for patients with recurrent EOC.

PD-1 is an immune checkpoint receptor expressed in cytotoxic T cells, which binds to PD-L1 in order to activate the inhibitory signal transduction in T cells, thereby mediating the immune escape of tumor cells.^[18]^ Pembrolizumab, a highly selective PD-1 inhibitor, has been approved by the FDA for the treatment of various cancers, including malignant melanoma, non-small cell lung cancer, and Hodgkin lymphoma. Several studies showed PD-1/PD-L1 inhibitors with therapeutic potential in ovarian cancer.^[19,20]^ A phase IB trial (KEYNOTE-028) included 26 patients with PD-L1-positive ovarian cancer has shown promising outcomes. One of the 26 enrolled patients achieved complete remission and 2 of patients showed partial remission.^[19]^ It was reported an ORR of 11.5% and the main adverse events were fatigue, anemia, and inappetence.^[19]^ In a phase II trial (KEYNOTE-010), another anti-PD-1 antibody, Nivolumab, was used in platinum-resistant patients with ovarian cancer. The ORR was 15% including 1 patient with partial response and two patients with complete responses, and the disease control rate was 45%.^[20]^ Recently, 2 case reports have demonstrated that pembrolizumab monotherapy or combination therapy is effective to control the progress of disease including metastatic ovarian carcinosarcoma and ovarian adenosquamous carcinoma.^[21,22]^ Additionally, some clinical trials have shown that pembrolizumab therapy is effective for other gynecologic cancers, including cervical and endometrial cancer.^[23,24]^ In May 2017, the FDA has approved pembrolizumab for patients with unresectable or MSI-H or mismatch repair gene defects (dMMR) solid tumors that progressed following prior treatment.

Although immunotherapy has made breakthroughs in multiple tumors, but the efficiency of immunotherapy still needs to be further improved. Recent studies have shown that the combination of immunotherapy and chemotherapy may have a synergistic effect in controlling tumors. Some chemotherapeutic agents with noncytotoxic concentrations can increase tumor immunogenicity, which can be used for cell-based anticancer immunotherapies.^[25]^ Herein, we sought to explore the effects of combination of pembrolizumab and nab-paclitaxel.

In this case, the patient was treated with multiple-line systemic therapies including surgery, multiple lines of chemotherapy, and anti-angiogenesis therapy. Liver damage, the main adverse event, was observed in the previous treatment, so long-term oral liver protecting drugs were required. However, in the combination of pembrolizumab and chemotherapy for the patient, the liver damage was not aggravated and no serious events were observed. Since the patient showed MSI-H, which is an effective predictor for PD-1/PD-L1 inhibitors therapies,^[26]^ and, for this reason, treated with pembrolizumab and chemotherapy showing the excellent therapeutic effects.

In conclusion, pembrolizumab combined with chemotherapy appeared to provide a positive antitumor effect in multiple metastatic lesions for this patient. We expect further data for the efficacy and safety of combination therapies including immunotherapies combined with chemotherapy, to provide evidence for the treatment of rare types of solid cancer.

## Author contributions

**Conceptualization:** Jian Jiang.

**Data curation:** Jian Jiang.

**Formal analysis:** Jian Jiang, Zhi-peng Chen.

**Funding acquisition:** Yun Zuo.

**Investigation:** Jian Jiang, Zhi-peng Chen, Xiao-lan Qian.

**Methodology:** Jian Jiang, Yong-qin Zhang, Min Zhang.

**Supervision:** Yun Zuo, Chen Ni, Hui-pin Zhu.

**Software:** Jian Jiang, Yun Zuo.

**Writing – original draft:** Jian Jiang, Zhi-peng Chen.

**Writing – review & editing:** Yun Zuo, Chen Ni.
